# An innovative technique of harvesting soil gas as a highly efficient source of ^222^Rn for calibration applications in a walk-in type chamber: part-1

**DOI:** 10.1038/s41598-020-73320-9

**Published:** 2020-10-06

**Authors:** N. Karunakara, Trilochana Shetty, B. K. Sahoo, K. Sudeep Kumara, B. K. Sapra, Y. S. Mayya

**Affiliations:** 1 Centre for Advanced Research in Environmental Radioactivity (CARER), Mangalore University, Mangalagangothri, Mangalore, 574 199 India; 2 Radiological Physics and Advisory Division, Bhabha Atomic Research Centre, Trombay, Mumbai, 400 085 India; 3 Department of Chemical Engineering, IIT-Bombay, Mumbai, 400 076 India

**Keywords:** Environmental sciences, Physics

## Abstract

The paper describes a novel technique to harvest ^222^Rn laden air from soil gas of natural origin as a highly efficient source of ^222^Rn for calibration applications in a walk-in type ^222^Rn calibration chamber. The technique makes use of a soil probe of about 1 m to draw soil gas, through a dehumidifier and a delay volume, using an air pump to fill the calibration chamber. ^222^Rn concentration in the range of a few hundred Bq m^−3^ to a few tens of kBq m^−3^ was easily attained in the chamber of volume 22.7 m^3^ within a short pumping duration of 1 h. A new technique referred to as “semi-dynamic mode of operation” in which soil gas is injected into the calibration chamber at regular intervals to compensate for the loss of ^222^Rn due to decay and leak is discussed. Harvesting soil gas has many important advantages over the traditional methods of ^222^Rn generation for calibration experiments using finite sources such as solid flow-through, powdered emanation, and liquid sources. They are: (1) soil gas serves as an instantaneous natural source of ^222^Rn, very convenient to use unlike the high strength ^226^Ra sources used in the calibration laboratories, and has no radiation safety issues, (2) does not require licensing from the regulatory authority, and (3) it can be used continuously as a non-depleting reservoir of ^222^Rn, unlike other finite sources. The newly developed technique would eliminate the need for expensive radioactive sources and thereby offers immense application in a variety of day to day experiments—both in students and research laboratories.

## Introduction

The worldwide recognition of ^222^Rn as a health hazard to the population has led to large scale environmental and indoor surveys aimed at evaluating concentration levels and dose assessments. To set-up standard protocols and maintain mutual conformity between the various detectors and instruments used by different laboratories, calibration facilities have been established in different countries^1–4^. It is desirable to have a large volume calibration facility for calibrating a large number of detectors simultaneously. Further large calibration chamber is preferred for calibrating ^222^Rn and ^222^Rn decay products monitors (and dosimeters) because the decay products tend to attach to walls of the chamber which may cause non-uniformity in the airborne decay products concentration in small chambers. An essential component of the calibration facility pertains to the availability of reliable radioactive source for injecting the ^222^Rn gas into the calibration chamber. The essential characteristics of such sources should be (1) capability to provide steady concentration for a long period, (2) free of interference from the ^220^Rn isotopes and decay products, (3) non-dependence of the source on environmental parameters, and (4) ability to deliver a wide range of concentration levels in the chamber. It is always advisable to calibrate the detector in the same measurement range for which it is designed. For example, several types of SSNTD based passive ^222^Rn dosimeter systems^5,6^ are being used in different indoor environments where concentration may vary significantly, from few tens to few thousands of Bq m^−3^, depending upon the ^222^Rn exhalation potential of soil and the building materials, and the ventilation rate. However, dosimeters used for these measurements are calibrated at a much higher level of concentration due to difficulty in maintaining low ^222^Rn concentration level in the calibration chamber for an extended period using the standard ^222^Rn sources.

Similarly, ^222^Rn measurement system used for high concentration measurements such as soil gas, Uranium tailings pond^7^ and Uranium mines needs to be calibrated at higher concentration levels. The calibration experiments performed worldwide in a walk-in kind of ^222^Rn chambers use different types of conventional sources such as solid flow-through type^2,4,8^, powdered emanation type^9,10^ and ^226^Ra solution type^11,12^ etc. One of the available standard sources often used by investigators is the well-known flow-through type ^222^Rn source (RN-1025, Pylon Electronics, Ottawa, Canada). This source is capable of meeting most of the requirements mentioned above, but with one limitation—it does not provide a steady ^222^Rn concentration when drawn for a long time duration. Hence, it is challenging to maintain the stability of concentration in a large calibration chamber. Thus, one looks for an alternate source of ^222^Rn delivery.

Variety of sampling techniques and measurement instrumentation used for ^222^Rn measurements require suitable calibration protocol and measurement standards. The measurement standards must be traceable to a reference ^222^Rn standard developed by the national reference standard generation body^13^. Establishing a primary ^222^Rn standard requires precise determination of the source activity and hence Uranium tailings and naturally enriched soils as ^222^Rn generators are avoided because of the uncertainties in their characterization and origin (NSRB-2018). In a secondary chamber such as the one described here, traceability is established with internal standard methods through the use of instruments which are calibrated in a primary calibration facility. The primary calibration facility is the one which has established traceability to NIST or another official standard laboratory. As a result of being equipped with the secondary measurement systems as a reliable tool, application of soil-gas as a secondary ^222^Rn standard for calibration experiments in a walk-in kind of calibration chamber is demonstrated in this paper.

The soil gas as a rich source of ^222^Rn and ^220^Rn and its use for calibration experiments have been demonstrated earlier by^14,15^ for small dimension chambers of volume 0.22 m^3^ and 0.13 m^3^ respectively. Soil gas was also used as a source of high ^222^Rn and ^220^Rn concentrations in studies aimed at developing techniques for mitigation of these gases in workplaces^16–18^. It was also used as a source of ^222^Rn for studying the ^222^Rn absorption capacity of edible oils^19^. But its use as a source for ^222^Rn in calibration and exposure experiments in walk-in kind of chambers has not been reported earlier, and the present work is first of its kind.

In this study, we demonstrate that temporal stability of ^222^Rn concentration in the chamber for detector exposures during calibration or inter-comparison experiments can be efficiently achieved through periodic injections of soil gas in semi-dynamic mode, in which ^222^Rn laden soil gas is pumped periodically^20^. Besides, we also demonstrate the application of soil gas for the static mode of operation of calibration chamber (in which the ^222^Rn concentration in the chamber is raised initially, and the chamber is then sealed, and experiments are conducted in the decay mode) for short term exposure of ^222^Rn detectors^21^.

### Advantages of soil gas over finite ^222^Rn source

As mentioned earlier, the application of a typical flow through radioactive sources of ^226^Ra (emanation type and solution type) for achieving a steady concentration of ^222^Rn in the calibration chamber has limitations. The operation of such radioactive sources is restricted to either (1) one-shot injection of built-in ^222^Rn to the chamber, or (2) steady-state mode of operation, in which ^222^Rn is drawn continuously and filled to the chamber. Finite ^226^Ra source cannot meet the requirement of supply of a steady ^222^Rn concentration over a long period since the built-in ^222^Rn activity is limited in these sources, and its generation rate is very slow. Both these modes of operation drain the ^222^Rn activity from source volume very rapidly, and its replenishment requires long time duration, a few days to up to a month, depending upon the strength of the source and ^222^Rn concentration desired for experiments. One can use multiple sources in the calibration facility, but this leads to cost escalation. Other disadvantages pertain to special arrangements required for storing the source (such as safe room and shielding requirements) and need for regulatory requirements from the national regulator to procure and operate the source. All the above problems are conveniently overcome by the use of naturally occurring soil as a steady source of ^222^Rn. The ^222^Rn concentration in soil gas of typical background radiation region, with ^226^Ra activity of 30 Bq kg^−1^ (worldwide average value), is expected to vary from 20 to 100 kBq m^−3^ depending upon the emanation factor, porosity and grain density^22^. In earlier publications, the use of soil gas as a steady source of ^222^Rn for small chamber experiments for a sufficiently long duration of up to 9 days was successfully demonstrated^14^. However, its applicability in large chambers and for a long period needs to be examined carefully. The ^222^Rn concentration in soil gas undergoes diurnal and seasonal variations which can be a limitation in the outset. One can overcome these by suitably regulating the flow rate and well-planned injection protocols. The details of adapting soil gas as a source of ^222^Rn for standard chamber experiments through improved delivery methods are discussed, and its advantages are delineated in this paper.

## Materials and method

### ^222^Rn calibration chamber at the Centre for Advanced Research in Environmental Radioactivity (CARER)

**Figure 1:**
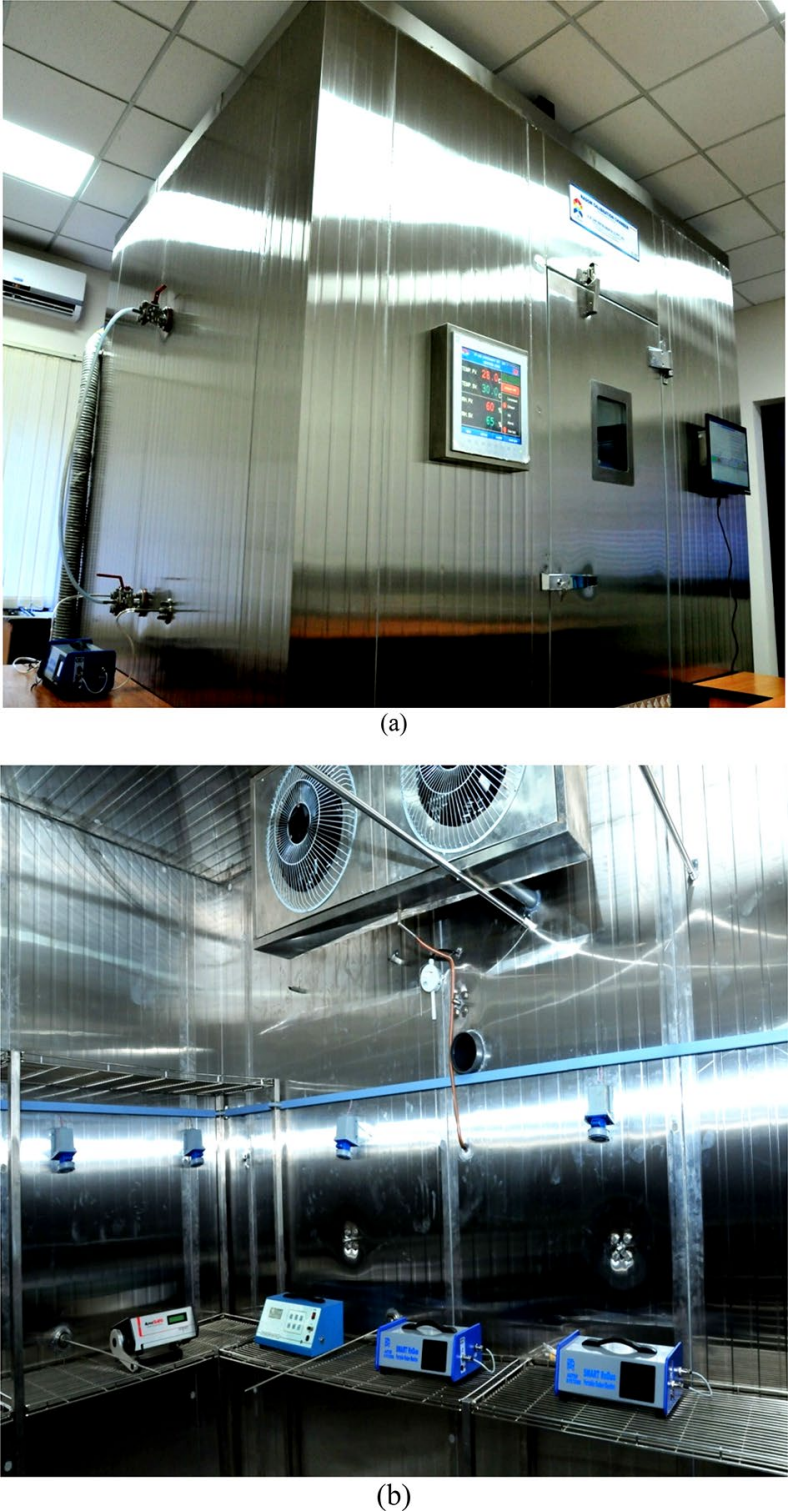
A view of the ^222^Rn calibration chamber at Centre for Advanced Research in Environmental Radioactivity (CARER) (**a**) exterior view, and (**b**) interior view.

The ^222^Rn calibration chamber installed at CARER is a walk-in type chamber, and its full exterior and interior views are shown in Fig. 1a, b respectively. The details of the design, construction, characterization and performance evaluation of the facility were published earlier^1^. Briefly, these aspects may be discussed as follows; the chamber has an internal air space volume of 22.7 m^3^ and is made of stainless steel with provisions for ^222^Rn injection, evacuation and sampling ports. Two mixing fans are operated with a combined airflow of 3620 m^3^ h^−1^ for homogenizing ^222^Rn concentration efficiently. Provisions are also available for deploying a large number of passive and several active ^222^Rn and progeny measuring devices simultaneously. The normal leak rates (mixing fan OFF condition) and the enhanced leak rates (mixing fan ON condition) were well characterized (to be 0.0011 h^−1^ and 0.0028 h^−1^ respectively) through a large number of ^222^Rn decay experiments^1^. Although in the present paper, we focus on the ^222^Rn related aspects of the calibration chamber, the chamber has been designed from the perspective of using it for ^222^Rn progeny monitoring device calibrations as well. The passive progeny detector, such as direct ^222^Rn progeny sensor (DRPS) and direct ^220^Rn progeny sensors (DTPS), needs to be calibrated in the environments similar to a typical dwelling. An essential characteristic of a typical dwelling is the surface area to volume ratio (s/v), which is ~ 2 m^−1^ as per international standard^23^. Hence, it is desirable that the calibration of the devices is performed in chambers with similar surface area to volume ratio and this forms the rationale for setting up a large chamber of volume 22.7 m^3^ (s/v ratio = 2.1 m^−1^) for ^222^Rn related experiments in the present study.

### Soil gas extraction and transport into the chamber

**Figure 2:**
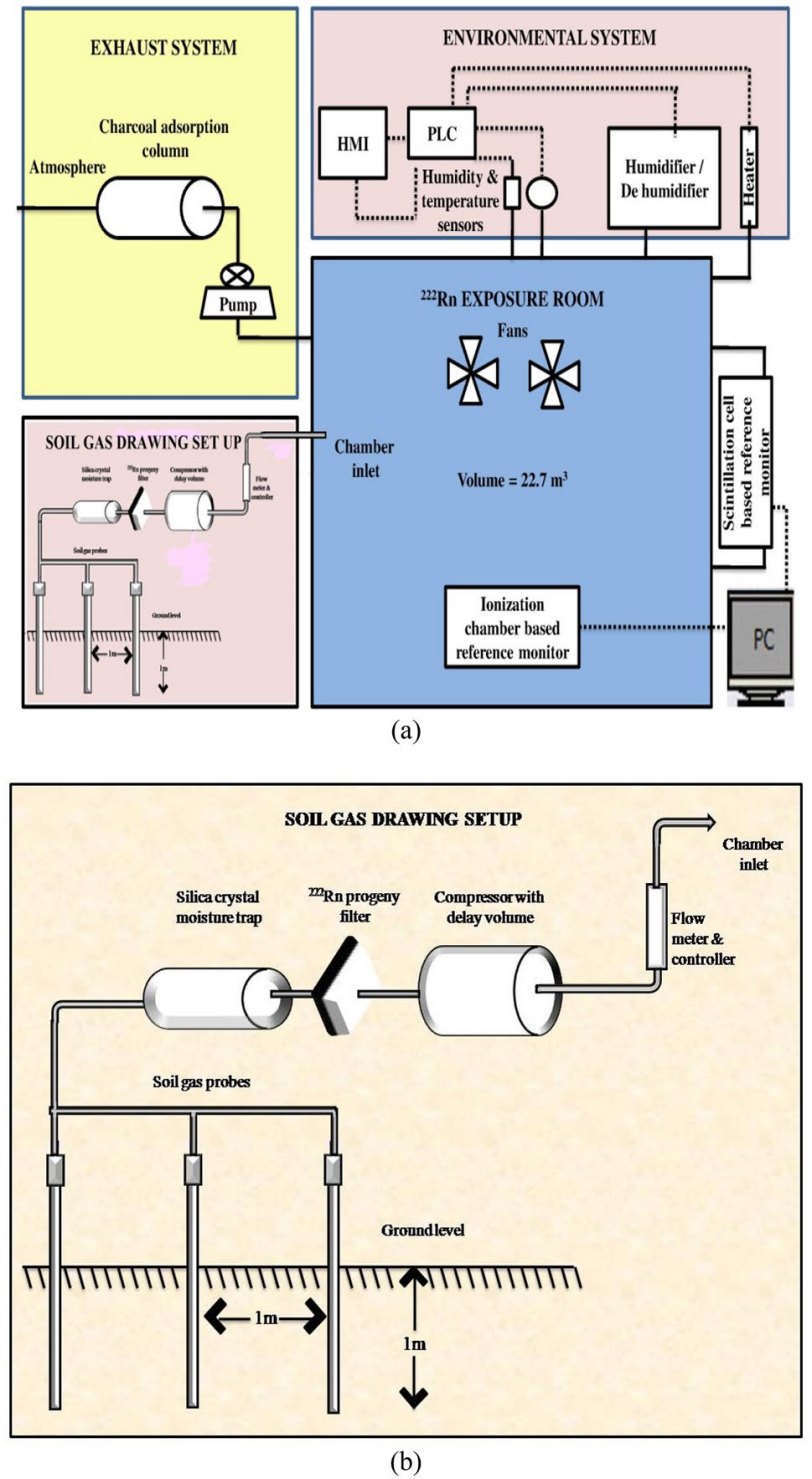
Schematic diagram of (**a**) calibration chamber and soil gas harvesting set-up (**b**) expanded view of soil gas harvesting set-up.

The ^222^Rn concentration in soil gas increases exponentially with depth and saturates to an equilibrium concentration at a depth of about 0.8–1 m^24,25^. The extraction was performed using a hand-driven soil gas probe (STITZ, Germany) inserted inside the ground to a depth of about 1 m, as shown in Fig. 2a, b^14,16^. The extraction was carried out at flow rates ranging from 10 to 80 L min^−1^, depending upon the experimental requirements. To avoid the possibilities of soil particles choking the inlet of the probes, the total flow was bifurcated into multiple channels, as shown in Fig. 2b. The bifurcation not only reduces the suction velocity at the probe inlet (thereby reducing the possibilities of soil particles blocking the probe inlet) but also reduces the possibility of large area perturbations of ^222^Rn concentration in the soil matrix. The outlets of multiple soil probes were combined and connected to a progeny filter, dehumidifier, air compressor, and a flow meter with a controller, which in turn was connected to the chamber inlet (Fig. 2b). The 0.052 m^3^ buffer volume of the air compressor serves as a stock for ^222^Rn and as a delay volume for mitigating ^220^Rn.

**Figure 3:**
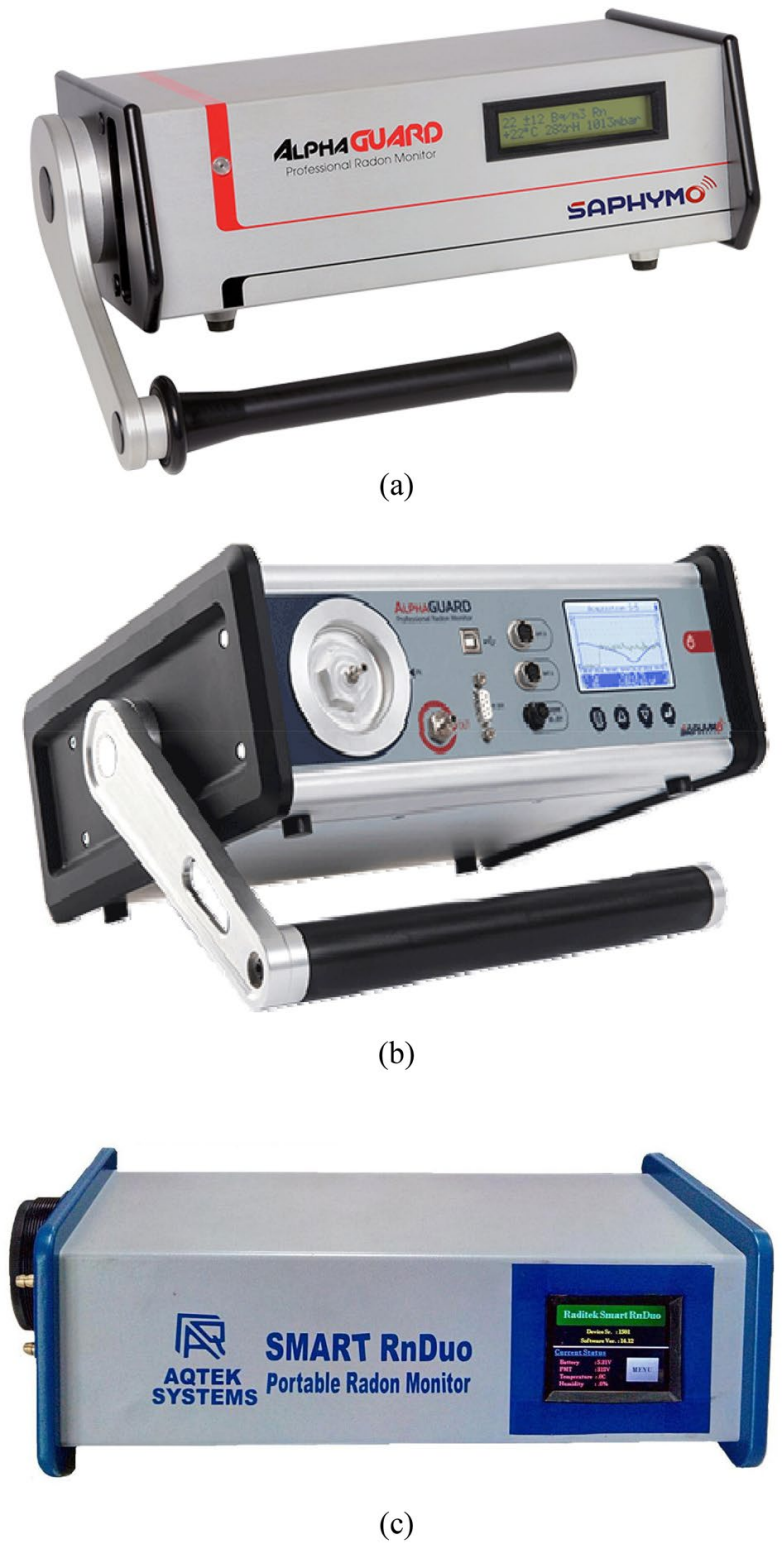
A view of the reference instruments used in the present study (**a**) AlphaGuard PQ2000PRO, (**b**) AlphaGuard Professional, and (**c**) Smart RnDuo.

### Reference ^222^Rn monitors

The reference instruments used for the present study are ionization chamber based monitors (AlphaGuard PQ2000PRO and AlphaGuard Professional, Saphymo, Germany), and scintillation cell-based monitors (Smart RnDuo, AQTEK SYSTEMS, India). A view of these instruments is presented in Figs. 3a, 3b and 3c. The detection range of ionization chamber based reference monitor is 2 Bq m^-3^ to 2000 kBq m^-3^, while that of scintillation cell-based monitor is 8 Bq m^-3^ to 50,000 kBq m^-3^. The details of these instruments were published earlier^1,14,16–19^. The primary calibration of the ionization chamber-based monitors was initially carried out under a Physikalisch Technische Bundesanstalt (PTB) reference atmosphere and that of scintillation cell-based monitors at BARC, Mumbai. In addition to this, both the type of reference monitors used here are periodically calibrated with an interval of a year at BARC, Mumbai using a standard solid flow-through type ^226^Ra source of activity 110.6 kBq (model RN -1025, Pylon electronics, Ottawa, Canada) with uncertainty < 2%. The readings provided by scintillation cell-based ^222^Rn monitors (Smart RnDuo) and ionization chamber based monitors (AlphaGuard) were found to match (within ± 1.1%) hence any of these instruments can be used as a reference monitor.

## Experiments, results, and discussion

Experiments were carried out to study two different aspects: characterization of soil gas as a source of ^222^Rn, and its application for generating desired ^222^Rn concentration in a large volume calibration chamber. The source characterization experiments were aimed to study (1) stability of ^222^Rn concentration when soil gas was drawn for long time duration and at different flow rates, (2) realizing temporal stability of ^222^Rn concentration in the chamber using soil gas, and (3) effective removal of ^220^Rn from the soil gas to ascertain its non-interference in the calibration experiments. Whereas, studies on the application of soil gas to generate desired ^222^Rn concentration in a large volume calibration chamber included the demonstration of (1) temporal stability in the ^222^Rn concentration in the chamber, (2) build-up characteristics of ^222^Rn in the chamber, (3) versatility of soil gas to achieve steady ^222^Rn concentration in the chamber, and (4) maximum values of ^222^Rn concentration attained in the chamber. These are discussed in the following sections.

### Characterization of the soil gas source

The technique of using soil gas is expected to work for most of the soils. The ^222^Rn concentration in the soil gas depends upon the ^226^Ra content of the soil, bulk density, emanation fraction and the moisture content and these parameters are related as given in Eq. (1)^22^:

1 $${\text{C}}^{{{\text{Rn}}}}_{{{\text{SG}}}} = {\text{C}}_{{{\text{Ra}}}} .{\text{f}}.\rho_{{\text{s}}} .{\epsilon}^{{ - {1}}} .({1} - {\epsilon}).\left( {{\text{m}}.\left[ {{\text{K}}_{{\text{T}}} {-}{ 1}} \right] \, + { 1}} \right)^{{ - {1}}}$$

where C^Rn^_SG_—^222^Rn concentration in soil gas (kBq m^−3^), C_Ra_—^226^Ra concentration of the soil (Bq kg^−1^), f—^222^Rn emanation fraction, ρ_s_—material density of soil (kg m^−3^), ε—total porosity of soil including both water and gas phases, m—water-filled fraction of porosity, K_T_—partition coefficient of ^222^Rn between water and gas phases.

As explained in the UNSCEAR report^22^ for a warm, moist soil (T = 25 °C, K_T_ = 0.23, m = 0.95) with typical soil parameters (C_Ra_ = 30 Bq kg^−1^, which is the worldwide average value for normal background radiation regions, f = 0.2, ε = 0.25) will have a concentration of ^222^Rn in pore air (C^Rn^_SG_) of 78 kBq m^−3^, which is 3.7 times higher than the value, for the same soil under cold and dry conditions (T = 0 °C, K_T_ = 0.53, m = 0.05, C^Rn^_SG_ = 21 kBq m^−3^). Concentrations below ~ 20 kBq m^−3^ are extremely unlikely in the soil gas, and this concentration would suffice to meet the requirements of the calibration experiments and thereby to make it potentially a credible natural source for any laboratory experiments.

The present study was conducted using the soil gas extracted from the lateritic soil prevailing in the premises of the laboratory at CARER, Mangalore University. The soil was characterized for its physical parameters such as the bulk density, seasonal moisture content, ^226^Ra and ^232^Th contents and ^222^Rn emanation power. Soil samples were collected at the soil gas harvesting location, and the concentrations of ^226^Ra and ^232^Th were measured by gamma spectrometry using High Purity Germanium (HPGe) detector (Canberra, USA) of 50% relative efficiency^26^. The details of the gamma spectrometer system used in the present study and technique for the analysis of radionuclide concentration in soil were published elsewhere^26–28^. Several measurements of surface exhalation rates of ^222^Rn and ^220^Rn were also performed for this site by chamber accumulation method^29,30^. The results of these measurements are presented in Table 1, along with other measured soil parameters. The mean values of surface exhalation rates were 39.0 ± 4.2 mBq m^−2^ s^−1^ and 130.0 ± 40.0 mBq m^−2^ s^−1^ for ^222^Rn and ^220^Rn respectively. These values are in agreement with the previously reported literature values for the location^31–33^. Also, physical properties of soil (such as ^222^Rn emanation power, the porosity of the soil, moisture content etc.) play an important role in governing the soil gas ^222^Rn concentration, in contrast, the soil ^226^Ra content does not explain this to a greater extent^33^.

**Table 1:** Radiological and physicochemical parameters of the soil.

Radiological and physicochemical parameters	Measured value
^222^Rn surface exhalation rate	39.0 ± 4.2 mBq m^−2^ s^−1^
^220^Rn surface exhalation rate	130.0 ± 40.0 mBq m^−2^ s^−1^
^40^ K activity concentration	185.0 ± 9.0 Bq kg^−1^
^226^Ra activity concentration	42.0 ± 1.0 Bq kg^−1^
^232^Th activity concentration	90.0 ± 2.0 Bq kg^−1^
Soil porosity (ε)	0.25
Emanation factor (f)	0.24
Soil grain density (ρ_S)_	990 kg m^−3^

From the measured data on ^226^Ra activity concentration, ^222^Rn emanation factor, soil grain density and total porosity of the soil (Table 1), the ^222^Rn concentration in the soil gas was calculated to be 109.0 kBq m^−3^ [Eq. (1)] for the location from where soil gas was drawn. We have considered a value of 0.95 for the water-filled fraction of porosity and 0.23 for the partition coefficient of ^222^Rn^22^. For comparison, the concentration of ^222^Rn in soil gas was measured in-situ as discussed in “Soil gas extraction and transport into the chamber” section, and the mean value was 99.0 ± 7.0 kBq m^−3^, which is in good agreement with the calculated value of 109 kBq m^−3^. This corresponds to an elevated category of classification range (100–250 kBq m^−3^) as described by^34^ for a soil gas mapping study in Germany.

#### Stability of ^222^Rn concentration in soil gas for long term operation

**Figure 4:**
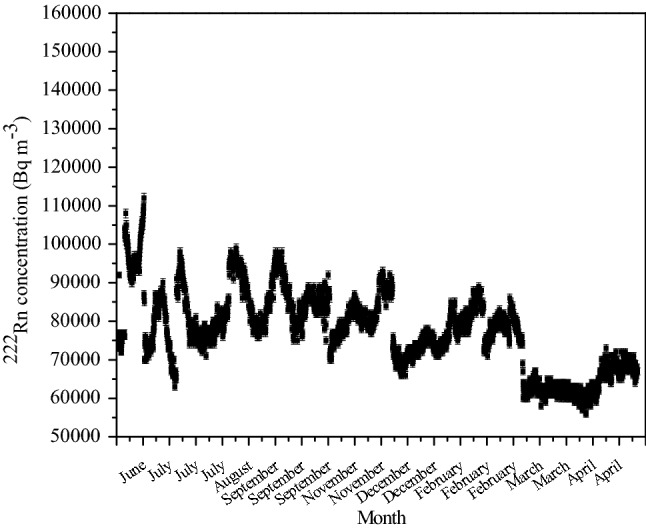
Variation of ^222^Rn concentrations in soil gas for 1 year (measured using scintillation cell-based online ^222^Rn monitor).

The stability of ^222^Rn concentration in soil gas was tested by harvesting it continuously during long term operation of over a month. In an earlier publication, we have demonstrated that soil gas technique is capable of providing steady ^222^Rn concentration when drawn at a flow rate of 10 L min^−1^^14,16^. We also draw attention to a previous study by one of these authors wherein ^222^Rn concentration was continuously measured for the duration of 30 days at a flow rate of 15 L min^−1^^35^. Similarly, monitoring of soil gas ^222^Rn concentration at the study site was performed at a flow rate of 1 L min^−1^ using a scintillation cell-based monitor for a year. An average concentration value of 78.0 ± 20.0 kBq m^−3^ (Fig. 4) was recorded, and the seasonal variation in concentration was within ± 20% of the annual average.

**Figure 5:**
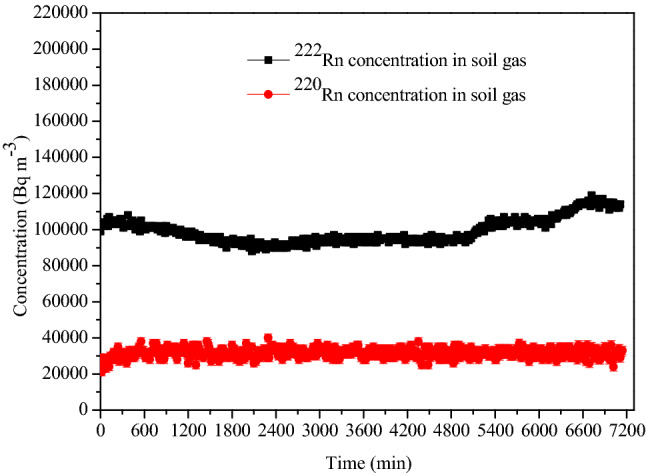
^222^Rn and ^220^Rn concentrations profiles in soil gas measured at the outlet of the soil probe.

To achieve the concentration of few kBq m^−3^ in a large calibration chamber in quick time, one needs to pump ^222^Rn laden air at a much higher flow rate. Therefore, we studied the variation of ^222^Rn concentration in the soil gas when drawn at a flow rate of 60 L min^−1^ for 7200 min and the monitoring was done continuously using scintillation cell-based monitors. The results (Fig. 5) confirmed that the concentrations remained steady with a mean value of ~ 99.0 ± 7.0 kBq m^−3^ for ^222^Rn and 32.0 ± 2.7 kBq m^−3^ for ^220^Rn. This demonstrates the utility of soil gas as a continuous and non-diluted source of ^222^Rn.

#### Removal of ^220^Rn from the soil gas

**Figure 6:**
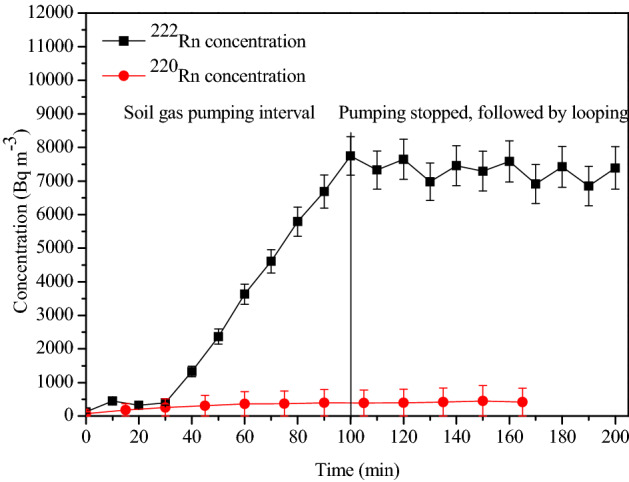
Concentration profiles of ^222^Rn and ^220^Rn in the chamber after pumping soil gas (^222^Rn measurements by AlphaGuard and ^220^Rn by Smart RnDuo).

In the next step, we demonstrate the effective removal of ^220^Rn from the soil gas so that the air stream entering the calibration chamber is free of it. As discussed in “Soil gas extraction and transport into the chamber” section (Fig. 2), the arrangement for harvesting the soil gas involved transporting it through a progeny filter, transport line of 20 m length, silica gel moisture trap containing ~ 2 kg of silica, air compressor with a delay volume of 0.05 m^3^, flow meter with the controller, and finally into the chamber inlet port. Experiments were conducted to confirm that during the transport of the soil gas from the soil to the chamber the delay provided by the transport line and the delay volume is adequate for the removal of ^220^Rn through radioactive decay (T_1/2_ = 55 s). The absence of ^220^Rn in the chamber air was confirmed for the highest flow rate of 80 L min^−1^ of soil gas, employed in the present study. The results of these studies are presented in Fig. 6, which demonstrates that the ^220^Rn concentration in the chamber is below the detector minimum detection limit (8 Bq m^−3^).

### Demonstration of the application of soil gas as a ^222^Rn source for calibration chamber

Upon ensuring the potential of the soil gas to serve as a convenient and un-depleted source of ^222^Rn, experiments were carried to harvest it for achieving desired concentration levels in the calibration chamber. The temporal stability and build-up characteristics of ^222^Rn concentration in the chamber were also studied, and details are presented in the following sections.

#### Achieving temporal stability in ^222^Rn concentration in the chamber

Calibration of measuring devices or any other exposure experiments are generally performed in the chamber under (1) static mode of operation^21^, or (2) dynamic mode of operation^21^. The static mode of operation is practically uncomplicated as it requires one-time filling of the chamber with ^222^Rn laden air to obtain desired concentration, and the experiments are performed during the decay of ^222^Rn. During the experiments, the ^222^Rn concentration in the chamber is measured continuously, and the average value is considered for evaluating the response of the detectors which are being examined.

On the other hand, in the dynamic mode of operation, the calibration experiments are performed under a stable ^222^Rn concentration environment (with deviations lesser than 10% from the reference concentration value) throughout the exposure duration^36,37^. Here the concentration stability is generally achieved by continuous pumping of ^222^Rn rich air from the source to the chamber throughout the experimental duration^21^. Dynamic mode of operation is the preferred method of calibration of devices because of the lower uncertainty associated it. However, this method has a disadvantage: it requires a ^226^Ra source of very high strength for continuous release of desired ^222^Rn activity throughout the experiment.

Hence, we demonstrate an innovative technique which we refer to as “semi-dynamic mode” of operation. As mentioned earlier, the decrease in the ^222^Rn concentration inside the chamber after filling it with the desired level is due to unavoidable processes such as radioactive decay and possible leak, as discussed in the section “^222^Rn calibration chamber at the Centre for Advanced Research in Environmental Radioactivity (CARER)”. These losses can be offset by a judicious combination of periodic injection of ^222^Rn laden air for a pre-set duration of pumping. In the new technique, pulsed injection of ^222^Rn at predetermined intervals was performed to compensate for its loss through decay and leak, as explained below.

**Figure 7:**
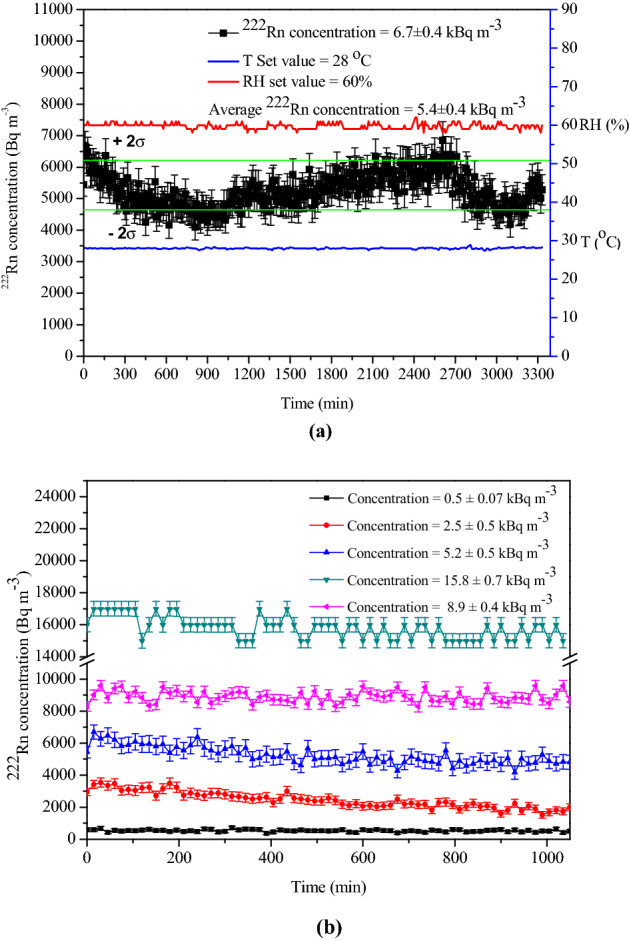
Illustration of the useful application of semi-dynamic mode of operation in combination with soil gas to achieve desired and steady-state ^222^Rn concentration in the chamber. (**a**) application of semi-dynamic mode of operation in which loss of ^222^Rn due to decay and leak is compensated with periodic injection, (**b**) demonstration of the versatility of soil gas harvesting technique to achieve desired steady**-**state ^222^Rn concentration levels in the calibration chamber.

Let us stipulate that it is required to maintain a steady concentration of 6 kBq m^−3^ with a maximum deviation of 10% (which happens to be the measurement uncertainty of any monitoring devices) for an operational period of 48 h. To accomplish this, the soil gas was pumped into the chamber at a flow rate of 30 L min^−1^ for 60 min. The concentration in the chamber builds up to a slightly higher value (6.7 ± 0.4 kBq m^−3^) than the target value (Fig. 7a). Now the pumping was stopped and the periodic injection protocol (pumping for 1 min in a period of 1 h) was adopted. This choice was based on the knowledge of chamber leak rate and ^222^Rn concentration in soil gas. The experiment was carried out for a duration of 48 h maintaining a relative humidity (RH) of 60% and a temperature (T) of 28 °C inside the chamber. The corresponding temporal variation of ^222^Rn in the chamber is shown in Fig. 7a and the excursions of the ^222^Rn profile are well within the previously stipulated bounds of 10%.

In Fig. 7b, we illustrate with more experimental results, the useful application of semi-dynamic mode of operation in combination with soil gas to achieve desired and steady-state ^222^Rn concentration in the chamber. Both low and high concentration values can be maintained in the chamber using the soil gas, as illustrated in Fig. 7b for 0.51 ± 0.073 kBq m^−3^, 2.5 ± 0.5 kBq m^−3^, 5.2 ± 0.5 kBq m^−3^, 8.9 ± 0.4 kBq m^−3^ and 15.8 ± 0.7 kBq m^−3^. It may be remarked that the choice of 1 min pumping in a period of 1 h, discussed just above, is rather empirical and was chosen merely to illustrate the technique. It is possible to arrive at an optimum pumping periodicity by a theoretical analysis of the ^222^Rn growth and decay equations and then adopt those optimized parameters in the experiments for achieving highly stable concentration value, as desired by the user, for the calibration application. With such a methodology, one can easily overcome the difficulties in maintaining concentration stability in the calibration chamber due to fluctuations of ^222^Rn concentration in soil gas. This was successfully attempted, and the details are presented in an adjoining publication, as Part-2 of this work^38^.

**Figure 8:**
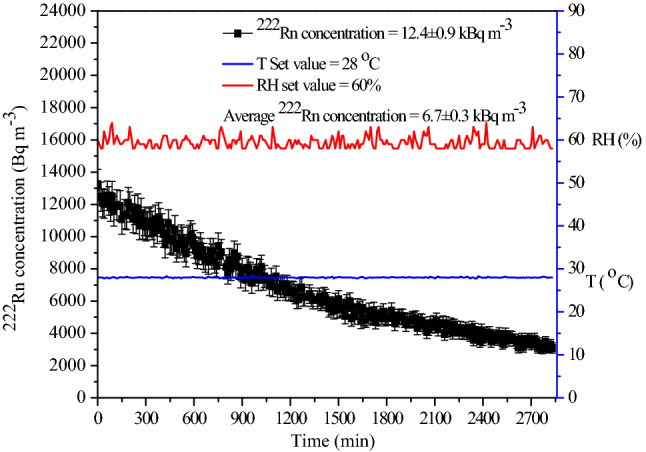
Illustration of the application of soil gas for the static mode operation of the chamber. One-time filling of the chamber with ^222^Rn and the experiments are performed during the decay of ^222^Rn.

The use of soil gas for the static mode of operation of the chamber, discussed above, was also studied. For an illustration, the soil gas was injected at a flow rate of 60 L min^−1^ for 60 min. Because of this, an initial ^222^Rn concentration of 12.4 ± 0.9 kBq m^−3^ in the chamber was attained. The concentration decay pattern was monitored continuously, and the results are shown in Fig. 8. The average value of concentration was observed to be 6.7 ± 0.3 kBq m^−3^ and, as stated earlier in this section, this value was considered for evaluating the response of the detectors under calibration exercise. It may be noted that this is a purely representative result, and one could start with the desired initial concentration by adequately injecting the soil gas to perform calibration experiments in a static mode of operation.

#### Build-up characteristics of ^222^Rn in the chamber

**Figure 9:**
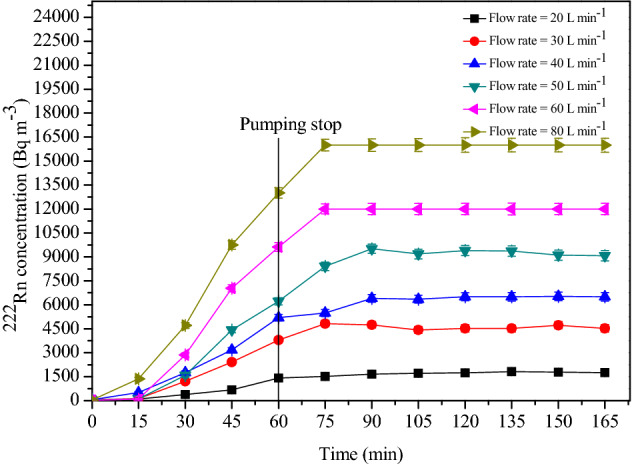
The build-up of ^222^Rn concentration inside the chamber at various injection rates for a fixed injection time of 60 min.

**Figure 10:**
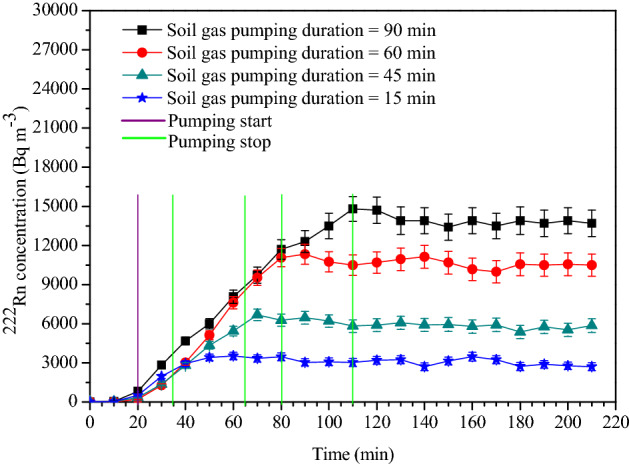
The build-up of ^222^Rn concentration achieved in the chamber for a fixed flow rate of 60 L min^−1^. Duration of injection varied from 15 to 90 min.

Experiments were conducted by injecting soil gas into the calibration chamber at different flow rates and durations to study the build-up characteristics of ^222^Rn concentration. Rapid mixing of ^222^Rn in the chamber was ensured by operating the mixing fans. The variation of ^222^Rn concentration in the chamber for different injection rates and duration of injection are presented in Figs. 9 and 10. As illustrated, concentration in the chamber reached a steady-state after about 15 min of stopping injection, which can be considered as the response time of the system. Steady-state values remained stable over a considerable length of time (~ 2 h) in the chamber because of low leak rates (mean T_1/2_ = 69 h). These steady-state values increased linearly with the volume of soil gas injected.

#### Versatility of soil gas to achieve steady ^222^Rn concentration in the chamber

Now we demonstrate the versatility of soil gas as a ^222^Rn source for attaining and sustaining desired ^222^Rn concentrations in the calibration chamber for the long-time operation. Separate experiments were carried out by pumping soil gas into the chamber at different flow rates of 20 L min^−1^, 30 L min^−1^, 40 L min^−1^, 50 L min^−1^, 60 L min^−1^, and 80 L min^−1^. The build-up of ^222^Rn inside the chamber was monitored continuously with a scintillation cell-based reference monitor Smart RnDuo. The readings provided by Smart RnDuo were verified by the use of AlphaGuard monitors as well.

**Figure 11:**
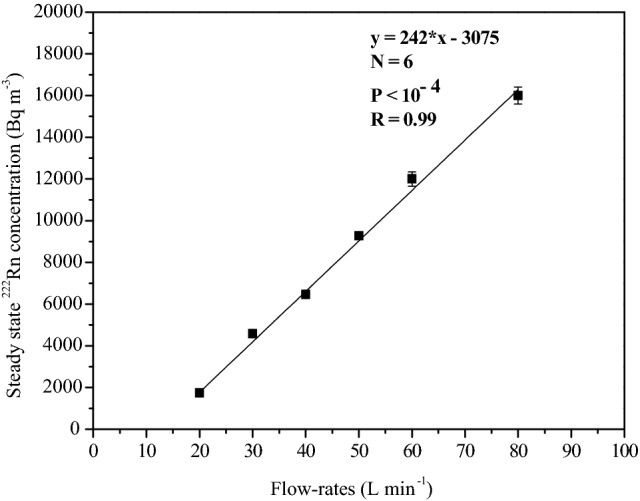
Correlation between the steady-state ^222^Rn concentrations attained in the chamber with the soil gas pumping rates (for a fixed pumping duration of 60 min).

As shown in Fig. 9, depending upon the injection rate, the concentration increased linearly with time, and when the injection was stopped, it attained a steady-state. The steady-state concentration value depends on the total volume of the soil gas injected, as shown in Figs. 9 and 10. The correlation plot of steady-state ^222^Rn concentration attained in the chamber with soil gas pumping rates for a fixed pumping duration of 60 min is presented in Fig. 11. A statistically significant positive correlation with R^2^ = 0.99 was observed between the two entities. An increase in the flow rate of soil gas by a factor of four induced an eight-fold increase in concentration in the chamber. The negative y-intercept indicates a decrease of ^222^Rn concentration in soil gas at low flow rates.

In addition to the actual measurements, one can calculate the expected concentration in the chamber with the knowledge of ^222^Rn concentration in soil gas and volume of soil gas pumped, using Eq. (2).

2 $${\text{C}}_{{{\text{ch}}}} = {\text{v}}.{\text{C}}^{{{\text{Rn}}}}_{{{\text{SG}}}} .{\text{V}}^{ - 1} \left( {{\text{kBq}}\;{\text{m}}^{ - 3} } \right)$$

where C_ch_—steady-state ^222^Rn concentration attained in the chamber (kBq m^−3^), v—volume of ^222^Rn laden air injected into the chamber (m^3^), C^Rn^_SG_—^222^Rn concentration in soil gas (kBq m^−3^) at the inlet of the chamber, V—chamber volume (m^3^).

**Figure 12:**
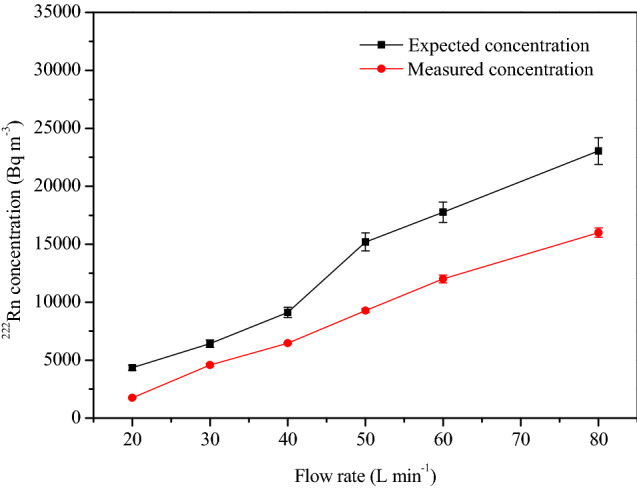
Comparison between calculated and measured concentrations in the chamber. The error bars shown with the expected concentration values were calculated by propagating the individual uncertainties associated with soil gas flow rate (± 4%) and soil gas ^222^Rn concentration (± 3%).

The steady-state concentrations, thus calculated for different flow rates, are compared with experimentally measured ones, as shown in Fig. 12. During these experiments, the RH content of the soil gas remained at ~ 90%. Whereas, the RH and T values in the chamber were maintained at 60% and 28 °C respectively. It is important to note that Eq. (2) assumes the ideal condition in which all the injected ^222^Rn is retained in the chamber. However, during experiments, there is loss due to leak and outflow from the chamber (as described in “^222^Rn calibration chamber at the Centre for Advanced Research in Environmental Radioactivity (CARER)” section). Because of this, the measured values are always lower than the calculated ones, particularly at higher flow rates.

#### Maximum values of ^222^Rn concentration attained in the chamber

**Figure 13:**
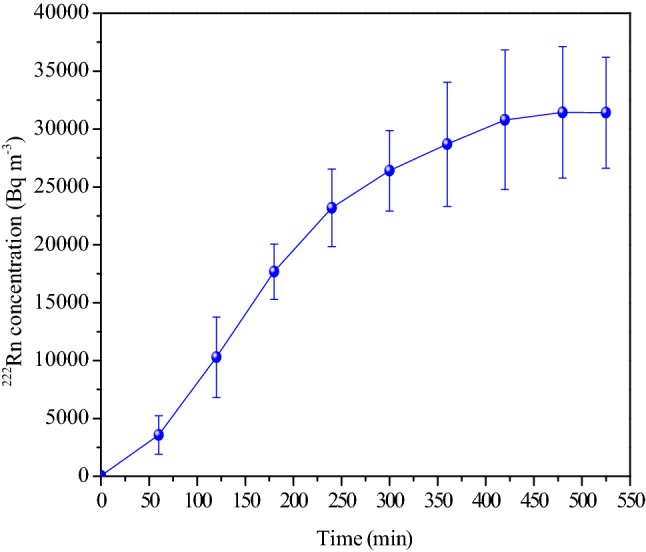
Maximum steady-state ^222^Rn concentration achieved in the chamber using a single soil gas probe (flow rate = 80 L min^−1^). The mean value of several measurements is plotted, and the error bar represents the deviation of individual result from the mean value.

The maximum ^222^Rn concentration that can be attained in the chamber using a single soil gas probe was also measured, and for this soil gas was continuously pumped into the chamber at a flow rate of 60 L min^−1^ until the steady-state concentration was achieved in the chamber. The time duration to attain this steady-state concentration was found to be ~ 7 h, and further pumping did not increase the concentration. The profile of concentration build-up is shown in Fig. 13. With a single soil gas probe, the highest steady ^222^Rn concentration that could be attained in the chamber (current chamber volume of 22.7 m^3^) was 31.4 ± 5.0 kBq m^−3^ (average value obtained from the readings recorded using three different monitors simultaneously). As pointed out earlier, ^222^Rn concentration at the outlet of the soil probe had a mean value of 99.0 ± 7.0 kBq m^−3^ when drawn continuously for 7200 min.

The above discussion makes it abundantly clear that the harvesting of ^222^Rn from soil gas has several advantages: (1) it is a non-diluting source, thereby providing the benefit of using it continuously for a long time duration, unlike conventional high activity ^226^Ra sources, (2) does not require elaborate shielding or special arrangements which are necessary for safe handling of conventional sources, and (3) ease in handling, free of cost, and does not require approval from the regulatory agencies.

## Comparison with other calibration chambers of the world

A comparison of important specifications and ^222^Rn sources used in some of the ^222^Rn calibration chambers available around the world are presented in Table 2. The large volume and small volume chamber facilities are grouped separately in this table. As summarised in Table 2 (column 5), other large volume calibration facilities, except the one described in the present study, make use of different types of ^226^Ra sources such as Pylon flow-through type, calcined ^226^Ra ceramic, lantern mantles, solid flow-through type- RF (multiple sources) or a combination of both dry and liquid sources, for ^222^Rn generation.

**Table 2:** Comparison of technical specifications for different ^222^Rn calibration chambers.

Institution (reference)	Chamber volume (m^3^)	Achievable ^222^Rn concentration range (kBq m^−3^)	Time to reach stable ^222^Rn concentration (h)	^222^Rn source	Mode of operation
**Large volume chambers**					
CARER, Mangalore University (present work)	22.7	0.5–31.0	0.5	Soil gas	Semi-dynamic
NIRS Japan^2,21^	25	0.5–8.0	12	Calcined ^226^Ra ceramic	Dynamic
NIM China^3^	20	1.0–232.0	Not specified	Solid flow through type- RF (multiple sources)	Semi-dynamic
Public Health England^21^	43	0.2–8.0	Static (one-shot injection)	Dry ^226^Ra source	Static
CLOR Warsaw-Poland^39^	16	3.7–50.0	6	Dry flow through Pylon- ^226^Ra	Static and dynamic
BFSGermany^21,40^	11, and 30	0.5–100.0	Static (one-shot injection)	Dry ^226^Ra source	Static
SURO Czech Republic^41^	45	0.1–100.0	Not specified	Dry ^226^Ra source	Static and dynamic
HPA-RPD England^42^	43	0.2–8.0	Not specified	Dry and liquid ^226^Ra source	Static
**Small volume chambers (< 1 m**^**3**^**)**					
BARC, Mumbai^5^	0.5	0.1–220.0	< 1 min	Pylon RN1025	Static
CARER, Mangalore University^14^	0.22	~ 80.0	< 10 min	Soil gas	Static
SURO Czech Republic^41^	0.15	Not specified	Not specified	Flow-through type mixed source	Static and dynamic
University of Huelva, Spain^43^	0.22	0.4–22.0	One-shot injection	Pylon RN 1025-20	Dynamic
Palermo University, Italy^44^	0.4	Not specified	Not specified	Dry ^226^Ra powder	Not specified
BFS^21,40^	0.4	0.5–100.0	Static (one-shot injection)	Dry ^226^Ra source	Static
Polish Academy of Sciences, Poland^45^	0.608	Up to 85.0	Not specified	Pylon^222^Rn	Static
Atomic Energy Commission, Syria^46^	0.65	Not specified	Not specified	Pylon source Model:2000	Not specified

As discussed in “Removal of ^220^Rn from the soil gas” section, in the dynamic mode of operation, ^222^Rn concentration gradually increases and attains a steady-state after some time. Time to reach steady-state concentration depends upon several factors such as type of source, its ^222^Rn generation rate, injection flow rate, the volume of chamber and leak rate of the chamber. The comparison highlights the fact that when dynamic method using ^226^Ra sources are employed, the time required to achieve stable concentration level in the chamber is very long, often higher by a magnitude when compared to that achieved by soil gas technique. It was also observed that irrespective of the mode of chamber operation, the concentration stabilization could be achieved very quickly using soil gas. In the majority of the calibration facilities static mode of operation was adopted^5,14,21^. However, this results in a short time exposure of the detectors due to the continuous decay of initially injected ^222^Rn with no scope for maintaining temporal stability.

## Conclusion

An innovative technique which uses ^222^Rn laden air from soil-gas of natural origin as a convenient and effective source of ^222^Rn for calibration applications in a large walk-in type calibration chamber has been developed and successfully demonstrated. Soil gas is a very stable source and is capable of providing steady non-depleting ^222^Rn concentration over a long period of operation. The desired ^222^Rn concentration in the chamber, from < 1.0 kBq m^−3^ to several tens of kBq m^−3^, can be attained very quickly and easily. Soil gas source has zero regeneration time and long-term stability when drawn continuously, unlike other radioactive sources of ^222^Rn. It serves as an instantaneous natural source of ^222^Rn, very convenient to use unlike the high strength ^226^Ra sources, has no radiation safety issues, and hence does not require any licensing from the regulatory authority. A semi-dynamic technique has been probed to maintain temporal stability of concentration by periodically pumping ^222^Rn into the calibration chamber. Approximate values for the injection rate, duration, and period were arrived at to compensate for the decay and leak of ^222^Rn from the chamber to maintain concentration stability within 10%. This study opens up an avenue for a rigorous theoretical analysis of the ^222^Rn growth and decay equations to arrive at optimum pumping parameters and adopt those parameters during the experiments for achieving desired and stable concentration in the chamber. This is presented in an adjoining publication, as Part-2 of this study series.
